# Benefits of inoculation, P fertilizer and manure on yields of common bean and soybean also increase yield of subsequent maize

**DOI:** 10.1016/j.agee.2017.08.015

**Published:** 2018-07-01

**Authors:** Edouard Rurangwa, Bernard Vanlauwe, Ken E. Giller

**Affiliations:** a Rwanda Agriculture Board, P.O. Box 5016, Kigali, Rwanda; b Plant Production Systems, Wageningen University, P.O. Box 430, 6700 AK Wageningen, The Netherlands; c Natural Resource Management Research Area, International Institute of Tropical Agriculture, P.O. Box 30772-00100, Nairobi, Kenya,

**Keywords:** Agro-ecological zone, Inoculation, Manure, P fertilizer, Yield

## Abstract

Common bean and soybean yield poorly on smallholder farms in Rwanda. We evaluated the benefits of inoculation combined with P fertilizer and manure on yields of common bean and soybean in three agro-ecological zones (AEZs), and their residual effects on a subsequent maize crop. In the first season, the treatments included inoculum, three rates of manure, and two rates of P fertilizer, with nine replications (three per AEZ). Both legumes responded well to inoculation if applied together with manure and P fertilizer. Grain yields varied from 1.0 t ha^−1^ to 1.7 t ha^−1^ in unamended control plots to 4.8 t ha^−1^ for common bean and 3.8 t ha^−1^ for soybean in inoculated plots with both P and manure addition. The response of common bean and soybean to inputs varied greatly between AEZs. In the AEZ with low and erratic rainfall (Bugesera), yields of both legumes and maize were low and maize after soybean failed to yield any grain due to drought. In this regard, early maturing legume varieties are advised in regions of low rainfall. Responses of maize to an input applied to the legumes strongly increased when other inputs were applied together to the legume. This allowed greater maize yields which ranged from 0.8 t ha^−1^ in control plots to 6.5 t ha^−1^ in treatments previously inoculated with P and manure added for maize grown after common bean and from 1.9 t ha^−1^ in control plots to 5.3 t ha^−1^ for maize grown after soybean. The amount of N_2_-fixed measured using the ^15^N-natural abundance method differed between the two legumes and varied between 15 and 198 kg N_2_ ha^−1^ for common bean and between 15 and 186 kg N_2_ ha^−1^ for soybean and differed enormously among treatments and AEZs. Application of inputs to the legumes also resulted in enhanced N and P uptake of the subsequent maize. The use of inoculum combined with manure and P fertilizer is a good option for smallholder farmers growing common bean and soybean in rotation with maize. We observed strong effects of environment and call for care when targeting crops and technologies for sustainable crop production.

## Highlights

• Yields and response to inputs of legumes and subsequent maize were strongest in sites with good rainfall.
• Rhizobium inoculation, P fertiliser and manure had synergistic effects on yield of soybean.
• Responses to treatments applied to legumes were reflected in increased yields of subsequent maize.
• At the driest site maize failed to yield due to the long maturity time of the preceding soybean variety.

## 1 Introduction

Legumes have an important role in improving soil health in sustainable agriculture (Vanlauwe et al., 2010). They have the ability, through symbiosis with rhizobia bacteria, to fix atmospheric nitrogen and yield well without mineral nitrogen fertilizer, improve soil fertility, and their rotation with cereals helps to control diseases and pests in cereals (Giller, 2001). However, the contribution of legumes to soil fertility is minimal if N_2_-fixation by the legume is constrained by an adverse environment (Giller and Cadisch, 1995). Integrated soil fertility management (ISFM) has gained much attention as a key option for boosting crop productivity through combining fertilizer use with other approaches to soil fertility management, adapted to local conditions (Vanlauwe et al., 2010). Various studies have shown the benefits of integrating ISFM in existing cropping systems. For instance, application of P fertilizer to the legume in a legume-maize rotation cropping system yielded high grain and biomass of the legume, which in turn resulted in better performance of the subsequent maize crop, thus reducing the need for external N fertilizer (Kihara et al., 2010; Vandamme et al., 2014). Targeting biological nitrogen fixation (BNF) technologies to agro-ecological niches within farming systems is of importance since the fertilizer is an expensive input which is hard to access for many smallholder farmers (Giller et al., 2013). If legume stover is not retained in the field, residual N is largely contributed by root and nodule senescence and fallen leaves (Ledgard and Giller, 1995). The benefits of legumes to the subsequent crops result not only from enhanced N availability following the legume crop but also from other rotational, non-N effects (Sanginga et al., 1999, Franke et al., 2016). These rotational effects include a reduction of pests and diseases, mobilization of poorly soluble P and increased mycorrhizal colonization of a subsequent cereal crop leading to enhanced P uptake (Bagayoko et al., 2000, Franke et al., 2016).

Population increase in Rwanda has led to small farm sizes, land fragmentation and soil fertility decline mainly as a result of intensive cropping with little or no nutrient inputs. The use of fallows to restore soil fertility is no longer possible (Rutunga et al., 1998). Common bean and soybean are the most widely cultivated legumes and promoted in the Rwandan Government’s Crop Intensification Programme (MINAGRI, 2009). The two legumes are grown for household consumption and for sale. Soybean cultivation is increasing due to its expanding market demand. Common bean is the main source of dietary protein: consumption was reported to be on average 38 kg of beans per person per year (CIAT, 2008). Yet, despite the high consumption of common bean and the expanding market demand of both beans and soybeans, yields achieved by smallholder farmers are poor: only 0.8–1.0 t bean ha^−1^ and 0.8–1.7 t soybean ha^−1^ (FAOSTAT, 2010).

Farmyard manure and mineral fertilizer are important options to increase crop productivity (Zingore et al., 2008a, Zingore et al., 2008b). Manure contribute not only to the restoration of soil fertility in depleted fields, but also in improving the response of crops to other nutrients, and enhanced N and P uptake in the legume-cereal rotations. As manure supplies exchangeable bases and other micronutrients, this helps to alleviate deficiencies reducing legume nodulation and N_2_-fixation. Despite the ‘One cow per poor family’ initiative which was introduced by the national government to boost agricultural productivity, the use of cattle manure in Rwanda is constrained by on-farm availability (MINAGRI, 2009). As elsewhere in Africa, the use of mineral fertilizers in Rwanda is limited by high costs (Kelly and Murekezi, 2000) and poor distribution systems (Vanlauwe and Giller, 2006).

Since indigenous rhizobia are not always in sufficient numbers, effective enough or compatible with the specific legume crop to stimulate BNF and increase yields, inoculation of legumes with rhizobia is an important option for enhancing BNF in crop production systems (Giller, 2001). The effectiveness of BNF is affected by agro-ecological factors. For instance poor nodulation and poor plant vigour in beans grown in soil with low extractable P led to a poor BNF (Amijee and Giller, 1998). However, if P fertilizer was added to beans, consistent responses to inoculation in BNF and grain yield were achieved. Other environmental stresses, such as high temperatures and dry soil can affect the symbiosis between common bean rhizobia, leading to a lack of responses to inoculation (Hungria et al., 2000).

Positive responses of cereal yields after the cultivation of legumes, relative to a cereal monoculture, have been reported frequently (Ojiem et al., 2014, Osunde et al., 2003, Franke et al., 2016). Yet we lack information on whether there are benefits of combined applications of inoculation with manure and/or P fertilizer application on the yields of grain legumes and whether these benefits are translated into increased yields of a subsequent cereal crop. We conducted a field study in three agroecological zones (AEZs) of Rwanda with the following objectives: (1) to assess the effect of inoculation, P fertilizer and manure addition on yield and yield components of common bean and soybean, (2) to evaluate the influence of environment on the response of the two legumes to inputs across the three AEZs, and (3) to evaluate how these treatments influence yield of a subsequent maize crop.

## 2 Materials and methods

### 2.1 Study sites

The study was carried out in farmers’ fields in three contrasting AEZs of Rwanda. In each AEZ, one district was selected where trials were established. Bugesera district was selected from the Bugesera AEZ, located in the South-East of the country at 02°12′18″S and 30°08′42″E at an altitude of 1435 m above sea level (masl), with a mean annual rainfall of 800 mm. Kamonyi district from the Granitic ridge AEZ, in the central plateau of the country, at 2°00′25″S and 29°50′49″E, 1661 masl, 1200–1400 mm rain. Kayonza district from the Eastern plateau AEZ in the eastern part of the country, at 1°55′59″S and 30°31′13″E, 1601 masl, 1000–1200 mm rain.

### 2.2 Trial establishment

Three experimental fields per district were selected for each legume in the short rains (SR) 2014 and maize was planted in the same treatments after the two legumes in the long rains (LR) 2014. In Bugesera and Kayonza, each treatment block with common bean was next to the one with soybean and blocks were replicated on three different farms in the same village. In Kamonyi, all three common bean treatment blocks were placed next to each other on the same farm, and two soybean blocks were placed on one farm, and the third block on another farm.

Three treatment factors applied to the legumes were: 1) without or with inoculation with *Rhizobium tropici* CIAT 899 for common bean and *Bradyrhizobium japonicum* USDA 110 for soybean; 2) manure at three rates: 0, 5 and 10 t ha^−1^; 3) P fertilizer at two rates: 0 and 30 kg P ha^−1^ added as triple super phosphate. The experiments were laid out in a split–split plot design with P fertilizer as the main plot, inoculum as sub-plot and manure as sub-sub-plot with a full set of treatments per block. Plot size was 5 m × 5 m. Next to each treatment block, a plot (5 m × 5 m) sown with maize served as a reference crop to assess BNF. The reference crop plots were fertilized with 5 t ha^−1^ of manure and weeds were controlled by hand. No P fertilizer was added to the reference crop.

The SR start in September and end in December, and the LR follow from March to June. Land was prepared with a hand hoe. Common bean variety RWR 2245 and soybean variety SB 24 were planted at a density of 50 cm × 10 cm for common bean and 40 cm × 10 cm for soybean with 1 m paths between main plots and sub plots to minimize cross-contamination. Manure applied to the experimental fields was provided by the participating farmers, and applied to her/his own field. In the LR 2014 season, maize variety ZM 607 was planted in all treatments at a density of 75 cm × 30 cm. No nutrients were added to the maize. No maize was planted at Kayonza as farmers mixed up the treatments during ploughing.

### 2.3 Measurements

#### 2.3.1 Common bean and soybean

Prior to planting, soil and manure samples were collected from each experimental block for chemical analysis. Soil sampling (0–20 cm) at nine points in each field were done following a W shape. The nine samples were combined, air-dried and passed through a 2 mm sieve. Moreover, samples from the manure provided by the participating farmers were collected and chemically analysed. In the legumes, biomass and nodulation were assessed at mid-podding. A small sub-plot of 0.5 m^2^ (leaving 0.5 m away from the plot border) was sampled. All plants were cut at ground level and fresh weight was determined. A sub sample was taken and weighed, sun dried, then oven dried at 65 °C to constant weight, and re-weighed for dry biomass yield determination. After cutting the biomass, the underground parts were gently uprooted, washed and nodule count was done by scoring 0–5 as follows: 0: No nodules; 1: <5 nodules; 2: 5–10 nodules; 3: 11–20 nodules; 4: 21–50 nodules, and 5: >50 nodules. Final grain and stover yields were determined at crop maturity by harvesting all pods from the net plots excluding the outer plant lines of both sides of the plot, and determining total fresh weight. A sub-sample was taken, weighed and sun-dried for several days and then threshed by hand. Grains were cleaned by winnowing and subsequently weighed and the moisture content was determined using an electronic moisture meter. The haulms were harvested by cutting them at ground level. Total fresh weight of the haulms was taken. Representative sub-samples of haulms from each plot were taken, sun-dried, then oven dried at 65 °C to constant weight. Grain yield is presented at 12.5% moisture content, stover (haulms + husks) at 0% moisture. After harvest, the residues remained in the field.

#### 2.3.2 Maize

Maize grain and stover yield was measured at crop maturity. All maize plants within the harvest area were cut excluding one row at each side of the plot and the first and the last maize plant of each row. Cobs were separated from stover and their fresh weights were determined. A sub-sample of stover and cobs was taken, and cobs were shelled. Cobs and stover samples were sun-dried and oven-dried at 65 °C to constant weight and re-weighed. Maize grain yields are presented at 14% moisture.

### 2.4 Plant analysis and measurements of nitrogen fixation

Common bean and soybean shoots, and maize stover and grain were ground and digested in hot H_2_SO_4_ and H_2_O_2_ (Parkinson and Allen, 1975). N and P concentrations in the digests were determined colorimetrically (Okalebo et al., 1993). N_2_-fixation was measured using the ^15^N natural abundance method (Unkovich et al., 2008). After drying and grinding the shoot samples, ^15^N content was determined using a stable isotope mass spectrometer (Thermo Scientific, Delta V Advantage Isotope Ratio MS Coupled through Conflo IV to Thermo Scientific Flash HT/EA, KU Leuven). The proportion of N derived from atmosphere (%Ndfa) and amount of N_2_-fixed for both legumes were calculated as follows (Unkovich et al., 2008):

%Ndfa = (δ^15^N ref-δ^15^N leg)/(δ^15^N ref-*B)* × 100

Where δ^15^N ref and δ^15^N leg are the ^15^N natural abundance (‰) in the non-fixing reference crops (maize for this study) and the fixing species. The smallest values of δ^15^N were used as the *B-*values and were −1.44 for common bean and −1.67‰ for soybean (Peoples and Craswell, 1992).Amount of N_2_-fixed = (%Ndfa × Total N legume)/100Where Total N legume is the%N in the legume plant times the dry biomass yield of the legume plant.

Net N input = Total amount of N_2_-fixed − Total amount of N removed in grain

The total amount of N_2_-fixed includes the N content in the below-ground parts, estimated at 30% of the amount of N_2_-fixed in the shoots (Unkovich et al., 2008). Since legume grains were not analysed, the N concentration in grain was estimated at 3.0% for common bean and 4.6% for soybean (Nijhof, 1987) and was multiplied with observed grain yield to obtain the total amount of N in grain.

Common bean and soybean P uptake was estimated as shoot P uptake determined at mid-podding, and maize N and P uptake was represented by the total amount of N and P in the aboveground parts (stover and grain) at harvest. Due to the large number of treatments and samples, nutrient concentrations and estimates of nitrogen fixation were made only in the 0 and 10 t ha^−1^ manure treatments.

### 2.5 Data analysis

Statistical analysis considered sites, fertilizer, inoculation and manure as fixed factors and replicates as random factors. Analysis of variance (ANOVA) was used to detect differences due to inputs and rotational effect in a split–split plot design using the GenStat 16th edition. The effect of different factors and their interactions were compared by computing the standard errors of difference (SED). Treatments means were compared using the least significant differences (LSD) at *P* ≤ 0.05.

## 3 Results

### 3.1 Rainfall distribution and sowing dates

**Fig. 1:**
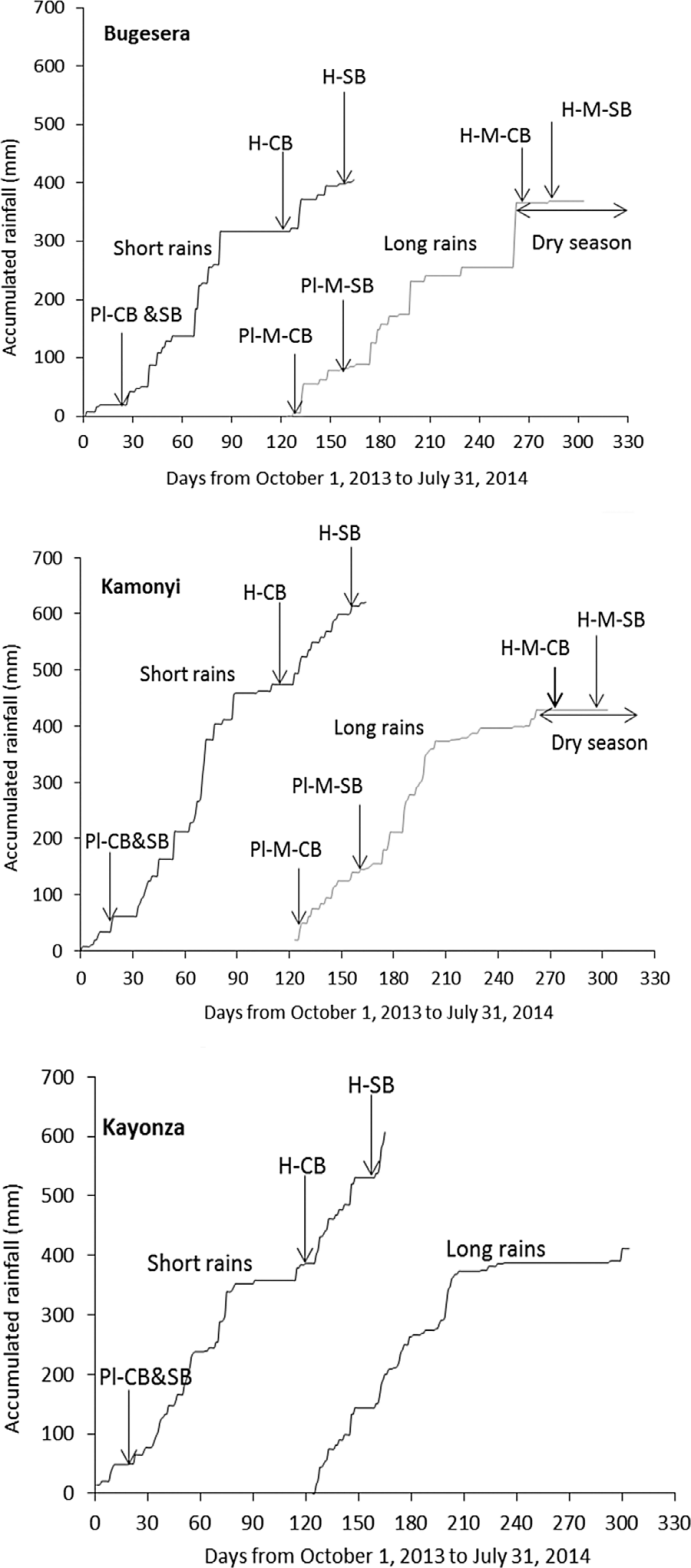
Rainfall distribution, sowing and harvesting dates at Bugesera, Kamonyi and Kayonza. Key: Pl = Planting; H = Harvest; CB = Common bean; SB = Soybean; M = Maize; No maize was planted at Kayonza in the long rains.

In all three AEZs, legume sowing was delayed by almost a month due to a late start of the rain season in 2013. Both legumes were sown on October 18, 2013 in Kamonyi, October 21 in Kayonza and October 23 in Bugesera. Common bean was harvested on January 23, 2014 in Kamonyi, January 28 in Bugesera and January 30 in Kayonza. Soybean was harvested a month later on March 4 2014 in Bugesera, March 6 in Kayonza and March 7 in Kamonyi. Maize after common bean was sown on February 4 2014 in Bugesera and February 5 in Kamonyi, while maize after soybean was sown on March 6 2014 in Bugesera and March 11 in Kamonyi. The maize variety took 141–146 days to mature, and the dry season in Bugesera started before maize sown after soybean was mature. Low rainfall, with dry spells in the middle of the season was observed during the LR. Bugesera received less and more poorly distributed rainfall (Fig. 1).

### 3.2 Soil and manure characteristics

Soil and manure samples collected before trial establishment differed across the AEZs (Table 1a). Soil pH was slightly acid to near-neutral. Soil available P varied greatly among the samples taken within each AEZ and was below the critical value of 10 mg P kg^−1^ in 12 out of 18 experimental blocks. The soil organic carbon in the three AEZs was above the reported critical value of 1.5% in all fields. Exchangeable cations were above the critical values of 0.2 for K and Mg, and 0.5 cmol_c_ kg^−1^ for Ca, so availability of these elements was unlikely to limit crop growth. The nutrient content of the manure (Table 1b) varied among the AEZs. The N concentration in manure from Bugesera (1.8%) was double that in manure from Kamonyi or Kayonza (0.9–1.0%). By contrast the largest P concentration was found in manure from Kamonyi. On average, 5 t of manure contained 90 kg N, 10 kg P and 70 kg K in Bugesera, 45 kg N, 25 kg P and 65 kg K in Kamonyi and 50 kg N, 10 kg P and 35 kg K in Kayonza.

**Table 1a:** Soil characteristics of experimental sites, averaged across each location.

Soil parameters	Bugesera		Kamonyi		Kayonza	
	Average	Range	Average	Range	Average	Range
pH (H_2_O)	6.2	5.0–7.5	6.2	5.5–6.5	6.2	5.4–6.6
Total N (g kg^−1^)	1.8	1.4–2.2	1.7	1.5–2.2	1.7	1.2–2.1
C (g kg^−1^)	24.1	16.3–31.4	20.2	16.4–24.8	25.6	18.6–32.5
P (Olsen) (mg P kg^−1^)	15.7	0.8–67.4	10.2	1.1–26.8	18.1	1.3–60.2
Exchangeable K (cmol_c_ kg^−1^)	0.4	0.1–0.9	0.8	0.3–1.3	0.6	0.1–0.9
Exchangeable Ca (cmol_c_ kg^−1^)	5.7	3.5–6.9	5.5	4.2–6.4	5.5	3.7–6.4
Exchangeable Mg (cmol_c_ kg^−1^)	1.8	0.8–2.4	1.8	1.4–2.1	1.9	1.2–2.3
ECEC (cmol_c_ kg^−1^)	14.4	5.8–20.0	11.1	8.4–17.9	15.7	6.1–25.0
Sand (g kg^−1^)	380	100–740	490	410–590	360	80–740
Silt (g kg^−1^)	120	70–200	120	50–180	130	90–180
Clay (g kg^−1^)	500	190–780	390	350–440	510	170–770

ECEC: Effective Cation Exchange Capacity.

**Table 1b:** Characteristics of the applied manure, averaged for each location.

Soil parameters	Bugesera		Kamonyi		Kayonza	
	Average	Range	Average	Range	Average	Range
pH (H_2_O)	8.7	8.3–9.3	8.2	7.7–9.0	8.5	7.5–9.6
C (%)	17.5	14.7–20.6	11.2	9.2–12.0	13.2	10.8–14.7
N (%)	1.8	1.6–2.1	0.9	1.0–1.4	1.0	0.7–1.6
P (%)	0.2	0.1–0.3	0.5	0.2–2.2	0.2	0.1–0.3
K (%)	1.4	0.7–2.5	1.3	0.7–1.5	0.7	0.3–1.3
Ca (%)	0.8	0.3–1.1	1.1	0.8–2.2	0.6	0.4–0.8
Mg (%)	0.4	0.2–0.5	0.9	0.3–1.4	0.3	0.1–0.4
S (%)	0.2	0.1–0.3	0.1	0.1–0.2	0.1	0.1–0.1
B (ppm)	43.4	22.0–48.4	40.9	20.3–52.0	43.6	18.5–57.4

### 3.3 Common bean and soybean yields

**Fig. 2:**
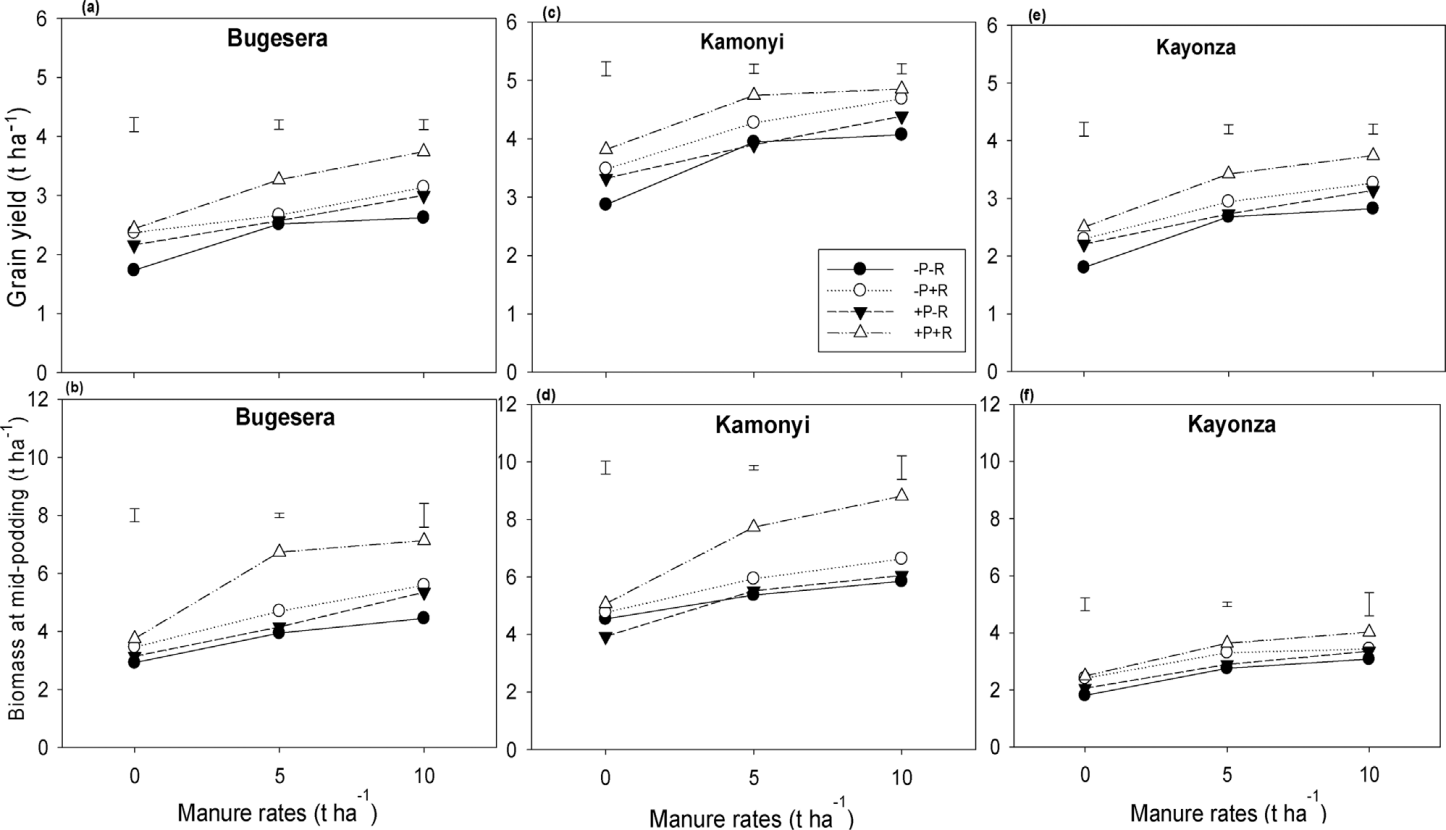
(a, c, e) Grain and (b, d, f) biomass at mid-podding yield response of common bean to inoculation, P fertilizer and three rates of manure at (a, b) Bugesera, (c, d) Kamonyi and (e, f) Kayonza. Error bars represent the standard errors of difference between means; −/+ R: without or with rhizobia (R) inoculation.

**Fig. 3:**
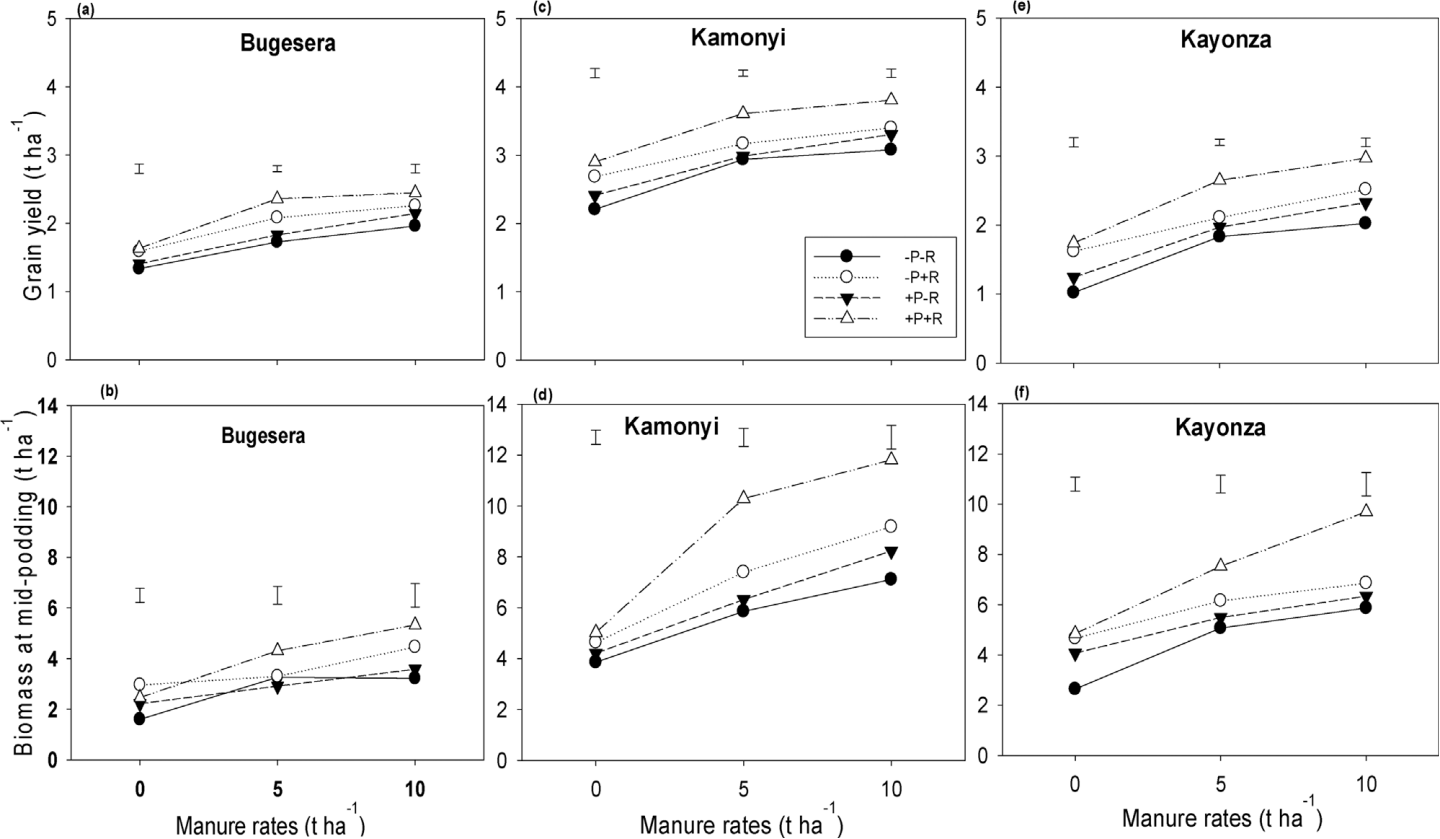
(a, c, e) Grain and (b, d, f) biomass at mid-podding yield response of soybean to inoculation, P fertilizer and three rates of manure at (a, b) Bugesera, (c, d) Kamonyi and (e, f) Kayonza. Error bars represent the standard errors of difference between means; −/+ R: without or with rhizobia (R) inoculation.

Grain and stover yield of common bean (Fig. 2, Table 2) and soybean (Fig. 3, Table 2) were greater in Kamonyi which received more and better distributed rainfall, though the differences were not significant. Small differences in biomass at mid-podding for both common bean (*P* = 0.073) and soybean (*P* = 0.019) were observed and biomass yield decreased with decreasing rainfall (Figs. 2 & 3).

**Table 2:** Stover response of common bean, soybean to inoculation combined with P fertilizer and three rates of manure at Bugesera, Kamonyi and Kayonza.

Treatments	Bugesera		Kamonyi		Kayonza	
	Common bean	Soybean	Common bean	Soybean	Common bean	Soybean
	Stover (t ha^−1^)	Stover (t ha^−1^)	Stover (t ha^−1^)	Stover (t ha^−1^)	Stover (t ha^−1^)	Stover (t ha^−1^)
0P-R + 0M	1.2	1.6	1.6	2.1	1.2	1.2
0P-R + 5M	1.6	1.8	2.4	2.9	1.5	1.7
0P-R + 10M	1.5	2.0	2.1	2.7	1.6	2.0
0P + R + 0M	1.5	1.9	2.0	2.5	1.4	1.5
0P + R + 5M	1.5	2.2	2.6	2.7	1.6	1.5
0P + R + 10M	1.7	2.4	2.6	2.9	1.9	2.0
30P-R + 0M	1.4	1.6	2.1	2.3	1.1	1.2
30P-R + 5M	1.4	1.9	2.1	2.4	1.5	1.6
30P-R + 10M	1.6	2.4	2.5	3.0	1.8	1.8
30P + R + 0M	1.2	1.8	2.0	2.4	1.4	1.8
30P + R + 5M	1.7	2.6	2.6	3.2	1.7	2.1
30P + R + 10M	2.0	2.7	2.6	3.0	2.1	2.4
Average	1.5	2.1	2.3	2.7	1.6	1.7
SED (Inoculum)	0.04	0.13	0.12	0.10	0.07	0.05
SED (Manure)	0.06	0.11	0.08	0.11	0.11	0.08
SED (Fert × Inoc × Manure)	0.08		0.18	0.22		0.15
SED (Fertilizer)			0.02			

Inputs of manure, inoculation and fertilizer significantly (*P <* 0.001) increased grain and stover yield, and biomass at mid-podding of both common bean and soybean, compared with unamended treatments across the three AEZs. Manure alone strongly increased the grain yield of common bean by 1.0 t ha^−1^. The response to manure application increased with inoculation and P fertilizer application to 1.2 t ha^−1^. Inoculation and P fertilizer increased the grain yield of common bean by 0.6 and 0.4 t ha^−1^ respectively. Although the overall effects of inoculation and P fertilizer were not significant, the combined treatment of inoculation and P together gave consistently the largest yield across all rates of manure at all three locations. The response of biomass at mid-podding to inoculation and P was strongest with the largest rate of manure. For instance in common bean, inoculation alone increased the biomass at mid-podding by 0.5 t ha^−1^, and the response to inoculation due to manure and P fertilizer was increased to 1.7 t ha^−1^. P fertilizer alone did not increase the biomass at mid-podding of common bean, but when added together with inoculation and manure gave an increase of 1.4 t ha^−1^. The largest rate of manure increased the biomass at mid-podding of common bean by 1.4 t ha^−1^, but together with inoculation and P fertilizer the response increased to 2.9 t ha^−1^. Similar trends were also observed for soybean (Fig. 3; Table 2). For both legumes, combined responses of all the three inputs together were greater than accumulated responses of single inputs for biomass at mid-podding. However, for grain and stover yields, synergistic effects of combined inputs were observed only for soybean and were not significant for common bean. For example in soybean, accumulated responses of single inputs was 4.9 and 1.4 t ha^−1^ against 6.2 and 1.6 t ha^−1^ achieved with combined responses of all inputs together for biomass at mid-podding and grain yield respectively.

### 3.4 Nodulation, nitrogen fixation, N and P uptake and net N input

**Fig. 4:**
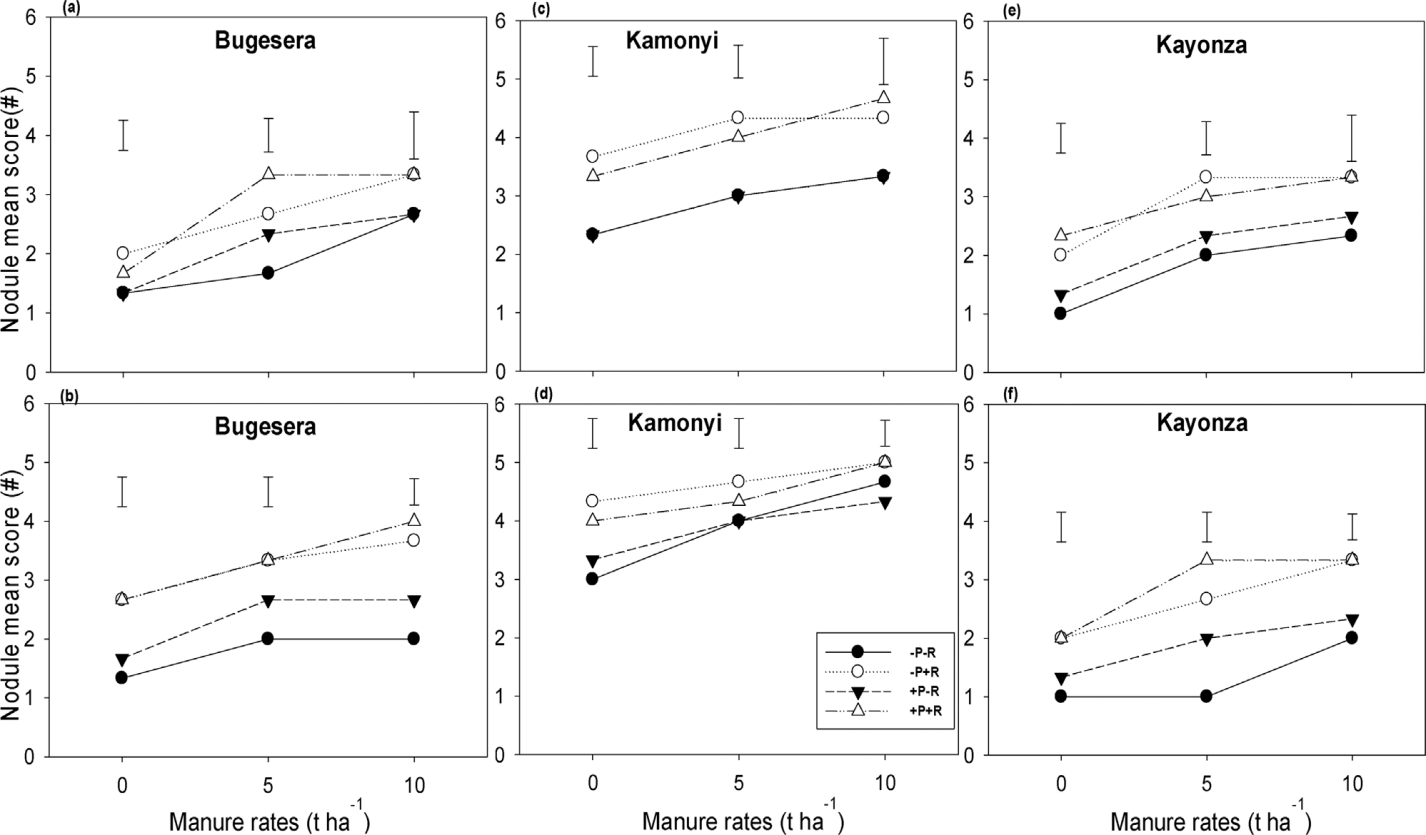
Nodule mean score (#) response: (a, c, e) common bean, and (b, d, f) soybean to inoculation, P fertilizer and three rates of manure at (a, b) Bugesera, (c, d) Kamonyi and (e, f) Kayonza. Error bars represent the standard errors of difference between means; −/+ R: without or with rhizobia (R) inoculation.

The number of nodules per plant in both common bean and soybean was assessed using nodule scores. Nodulation score significantly differed (*P <* 0.001) among the three AEZs with Kamonyi having the highest nodule score for both legumes. The nodule score of both common bean and soybean in the three AEZs increased with inoculation and increasing rate of manure (Fig. 4). There was no clear effect of P fertilizer on nodulation of both legumes.

Common bean generally fixed a smaller proportion of its nitrogen than soybean (Table 3, Table 4). The %Ndfa in common bean differed (*P* = 0.003) among the three AEZs and was on average largest in Kamonyi (53%) and least in Bugesera (24%). The%Ndfa in soybean was not affected by AEZ (*P* = 0.317). Surprisingly, inoculation had no significant effect on%Ndfa for either legume. Although 10 t ha^−1^ of manure often led to a smaller mean%Ndfa compared with unmanured treatments this difference was not significant.

**Table 3:** Common bean shoot δ^15^N, percentage of N_2_ derived from atmosphere (Ndfa), amount of N_2_-fixed and Net N input as affected by treatments.

AEZs/Fertilizer (kg ha^−1^)	Inoculum	Manure (t ha^−1^)	δ^15^N ref (‰)	shoot δ^15^N (‰)	Range shoot δ^15^N (‰)	%Ndfa	Total Amount N-fixed (kg ha^−1^)	Total N in grain (kgha^−1^)	Net N input (kg ha^−1^)
Bugesera									
0P	−R	0M	7.1	4.7	4.3–5.0	27	22.2	52.0	−29.8
0P	−R	10M	7.1	5.8	5.6–6.1	14	17.1	78.7	−61.6
30P	+R	0M	7.1	4.8	3.4–6.8	27	26.5	73.1	−46.7
30P	+R	10M	7.1	4.8	3.6–6.7	27	64.9	112.3	−47.5
Average/AEZ				5.0		24	32.7	79.0	−46.4

Kamonyi									
0P	−R	0M	11.4	3.8	1.6–7.6	53	84.6	86.1	−1.6
0P	−R	10M	11.4	5.1	4.2–6.5	45	105.3	122.3	−17
30P	+R	0M	11.4	3.4	2.5–3.9	62	117.7	114.5	3.2
30P	+R	10M	11.4	4.1	3.5–5.2	55	197.7	145.6	52.0
Average/AEZ				4.1		53	126.3	117.1	9.2

Kayonza									
0P	−R	0M	8.4	4.1	−1.4 to 7.5	43	18.2	54.1	−35.9
0P	−R	10M	8.4	7.6	6.9–8.2	12	14.5	84.6	−70.1
30P	+R	0M	8.4	5.4	4.7–6.2	33	34	75.1	−41.1
30P	+R	10M	8.4	5.4	4.8–6.1	33	55.3	112.3	−57.0
Average/AEZ				5.6		30	30.5	81.5	−51.0
SED (Fertilizer × Inoculum × Manure)				0.89		9.27	13.5	5	16.66
SED (AEZ × Manure × Inoculum)				1.09		11.35	16.54	6.12	20.41
SED (AEZ × Fertilizer × Inoculum)				1.09		11.35	16.54	6.12	20.41
SED (AEZ × Fertilizer × Inoculum × Manure)				1.54		16.06	23.39	8.66	28.86

−/+ R: without or with rhizobia (R) inoculation; trt: Treatment; ref: Reference.

**Table 4:** Soybean shoot δ^15^N, percentage of N_2_ derived from atmosphere (%Ndfa), amount of N_2_-fixed and Net N input as affected by treatments.

AEZs/Fertilizer (kg ha^−1^)	Inoculum	Manure (t ha^−1^)	δ^15^N reference (‰)	Shoot δ^15^N (‰)	Range Shoot δ^15^N (‰)	%Ndfa	Total amount N-fixed (kg ha^−1^)	Total N in grain (kg ha^−1^)	Net N input (kg ha^−1^)
Bugesera									
0P	−R	0M	8.0	3.2	−0.2 to 5.4	46	25.0	61.4	−36.4
0P	−R	10M	8.0	5.2	4.0–6.7	28	14.9	90.2	−75.3
30P	+R	0M	8.0	4.2	3.0–6.0	39	28.4	75.1	−46.7
30P	+R	10M	8.0	5.3	4.1–7.5	28	50.4	112.6	−62.3
Average/AEZ				4.5		35	29.7	84.8	−55.2

Kamonyi									
0P	−R	0M	8.4	3.7	0.3–6.7	44	71.0	101.5	−30.6
0P	−R	10M	8.4	2.1	1.2–3.8	62	184.3	141.6	42.8
30P	+R	0M	8.4	2.8	0.1–5.4	54	117.8	133.5	−15.6
30P	+R	10M	8.4	3.8	0.0–6.1	43	186.0	175.0	11.0
Average/AEZ				3.1		51	139.8	137.9	1.9

Kayonza									
0P	−R	0M	8.0	0.9	−1.7 to 5.1	69	58.5	47.0	11.5
0P	−R	10M	8.0	5.8	5.0–6.3	21	50.1	93.1	−43.0
30P	+R	0M	8.0	4.8	2.4–6.3	32	56.3	80.1	−23.8
30P	+R	10M	8.0	5.5	3.9–6.6	24	90.8	136.6	−45.8
Average/AEZ				4.3		37	63.9	89.2	−25.3
SED (Fertilizer × Inoculum × Manure)				0.95		12.76	19.52	5.05	19.86
SED (AEZ × Manure × Inoculum)				1.17		15.62	23.9	6.19	24.32
SED (AEZ × Fertilizer × Inoculum)				1.17		15.62	23.9	6.19	24.32
SED (AEZ × Fertilizer × Inoculum × Manure)				1.65		22.1	33.81	8.75	34.4

The amount of N_2_-fixed was on average larger in soybean than common bean (Table 3, Table 4). For both legumes, the largest amount of N_2_-fixed was observed at Kamonyi which received more and better distributed rainfall and had greater biomass production, and least at Kayonza for common bean and Bugesera for soybean. Inoculation combined with P fertilizer led to increased amount of N_2_-fixed by common bean and soybean, which was consistently more when combined with manure. Averaged over the three AEZs, inoculation combined with 30 kg P ha^−1^ increased the amount of N_2_-fixed by common bean by 17 kg N ha^−1^ over the control and by 64 kg N ha^−1^ when manure was added at 10 t manure ha^−1^. Similarly, inoculation combined with 30 kg P ha^−1^ increased the amount of N_2_-fixed by soybean by 16 kg N ha^−1^ without manure addition and by 57 kg N ha^−1^ when manure was added at 10 t manure ha^−1^.

Shoot N and P uptake by common bean significantly differed (*P <* 0.001) among the three AEZs (Table 5). For soybean, significant difference (*P <* 0.001) between the three AEZs was observed in shoot N uptake but less strong differences (*P* = 0.045) in P uptake. A greater mean shoot N and P uptake was observed at Kamonyi for both legumes and the least uptake at Kayonza for common bean and Bugesera for soybean shoot N uptake. Both legumes had a greater shoot N and P uptake in treatments that received full inputs and least in unamended treatments. For both legumes and in all AEZs, manure addition either alone or in combination with inoculation and P fertilizer, strongly and consistently enhanced N and P uptake (Table 5). N and P uptake in treatments that received inoculation and P fertilizer were also small when no manure was added. For instance, inoculation combined with 30 kg P ha^−1^ increased mean shoot N uptake in common bean by 19 kg N ha^−1^ over the control without manure addition and by 115 kg N ha^−1^ when manure was added at 10 t ha^−1^. Shoot N uptake in soybean increased as well with inoculation when combined with P fertilizer by 43 kg N ha^−1^ over the control without manure addition and by 193 kg N ha^−1^ with manure addition at 10 t ha^−1^. Shoot P uptake in both legumes was less affected by inoculation when no manure was added. For example, inoculation combined with 30 kg P ha^−1^ increased shoot P uptake in common bean by 3 kg P ha^−1^ without manure and by 16 kg P ha^−1^ when manure was added at 10 t ha^−1^. Similarly, inoculation and P fertilizer applied to soybean increased shoot P uptake by 4 kg P ha^−1^ without manure, and increased by 25 kg P ha^−1^ when manure was added (Table 5).

**Table 5:** Shoot N and P uptake (kg ha^−1^) of common bean and soybean as influenced by treatments.

Treatments	Bugesera AEZ				Kamonyi AEZ				Kayonza AEZ			
	Common bean		Soybean		Common bean		Soybean		Common bean		Soybean	
	Shoot P uptake	Shoot N uptake	Shoot P uptake	Shoot N uptake	Shoot P uptake	Shoot N uptake	Shoot P uptake	Shoot N uptake	Shoot P uptake	Shoot N uptake	Shoot P uptake	Shoot N uptake
0P-R + 0M	8	59	4	36	23	121	14	109	4	51	6	70
0P-R + 10M	12	119	21	45	28	157	26	213	9	89	11	169
30P + R + 0M	11	77	7	54	27	134	19	147	6	77	10	144
30P + R + 10M	29	200	23	144	44	254	53	338	10	122	24	312
Average	15	114	14	70	31	167	28	202	7	85	13	174
SED (Inoculum)	2.41	13.02	ns	17.27	2.41	13.02	ns	17.27	2.41	13.02	ns	17.27
SED (Fertilizer)	2.41	13.02	ns	17.27	2.41	13.02	ns	17.27	2.41	13.02	ns	17.27
SED (Manure)	2.41	13.02	5.19	17.27	2.41	13.02	5.19	17.27	2.41	13.02	5.19	17.27
SED (AEZ)	2.96	15.94	6.35	21.15	2.96	15.94	6.35	21.15	2.96	15.94	6.35	21.15

The net N input ranged widely from negative to positive for both common bean and soybean without any clear pattern or significant differences between treatments. A more positive net N input was observed in Kamonyi which received more and better distributed rainfall, where both legumes fixed a larger amount of N_2_ compared to more negative net N inputs observed in AEZs which experienced periods of dry spells (Table 3, Table 4). The net N input was strongly influenced by the amount of N_2_-fixed, the total N in grain and AEZ.

### 3.5 Nutrient uptake and production in maize

Maize accumulated more N and P when grown after common bean and soybean at Kamonyi than at Bugesera (*P <* 0.001; Table 6). The preceding legume also influenced N and P uptake by maize, with more N uptake achieved when maize was grown after common bean and more P uptake achieved when maize was grown after Soybean. In all cases, greater uptake was achieved in treatments that previously had received full inputs compared with previously unamended plots or plots that had received inoculation and P fertilizer without manure addition. Inoculation combined with 30 kg P ha^−1^ applied to common bean, on average, increased N uptake of the subsequent maize by 27 kg N ha^−1^ over maize uptake after unamended common bean and by 64 kg N ha^−1^ when manure was added at 10 t ha^−1^. Similarly, inoculation with P fertilizer applied to soybean, on average, increased N uptake of the subsequent maize by 16 kg N ha^−1^ over maize uptake when grown after untreated soybean, and by 52 kg N ha^−1^ when manure was added at 10 t ha^−1^. Maize P uptake grown after both common bean and soybean was increased by inputs applied to the two legumes and followed similar trend as N uptake (Table 6).

**Table 6:** Total N and P uptake (kg ha^−1^) of maize grown after common bean and soybean with and without inoculation, P fertilizer and manure.

Treatments	Bugesera AEZ				Kamonyi AEZ			
	Common bean-Maize		Soybean-Maize		Common bean-Maize		Soybean-Maize	
	Total P uptake	Total N uptake	P uptake	N uptake	Total P uptake	Total N uptake	Total P uptake	Total N uptake
0P-R + 0M	2	33	4	24	16	98	18	69
0P-R + 10M	3	68	5	24	23	119	35	68
30P + R + 0M	2	75	8	47	21	111	30	78
30P + R + 10M	10	116	9	64	21	144	55	134
Average	4	73	7	40	20	118	35	87
SED (Inoculum)	n.s	n.s	n.s	n.s	n.s	n.s	6.04	4.62
SED (Fertilizer)	n.s	n.s	n.s	n.s	n.s	n.s	6.04	4.62
SED (Manure)	n.s	15.58	n.s	n.s	n.s	15.58	6.04	4.62
SED (AEZ)	3.34	15.58	n.s	n.s	3.34	15.58	6.04	4.62
SED (Inoculum × manure)	n.s	n.s	n.s	n.s	n.s	n.s	n.s	6.54

Common bean-Maize: Maize grown after common bean; Soybean-Maize: Maize grown after soybean; ns: not significant at p < 0.05; 0 M/10 M: 0 and 10 t ha^−1^ of manure; P and N uptake by maize after soybean at Bugesera was calculated from stover only since maize did not yield any grain.

**Fig. 5:**
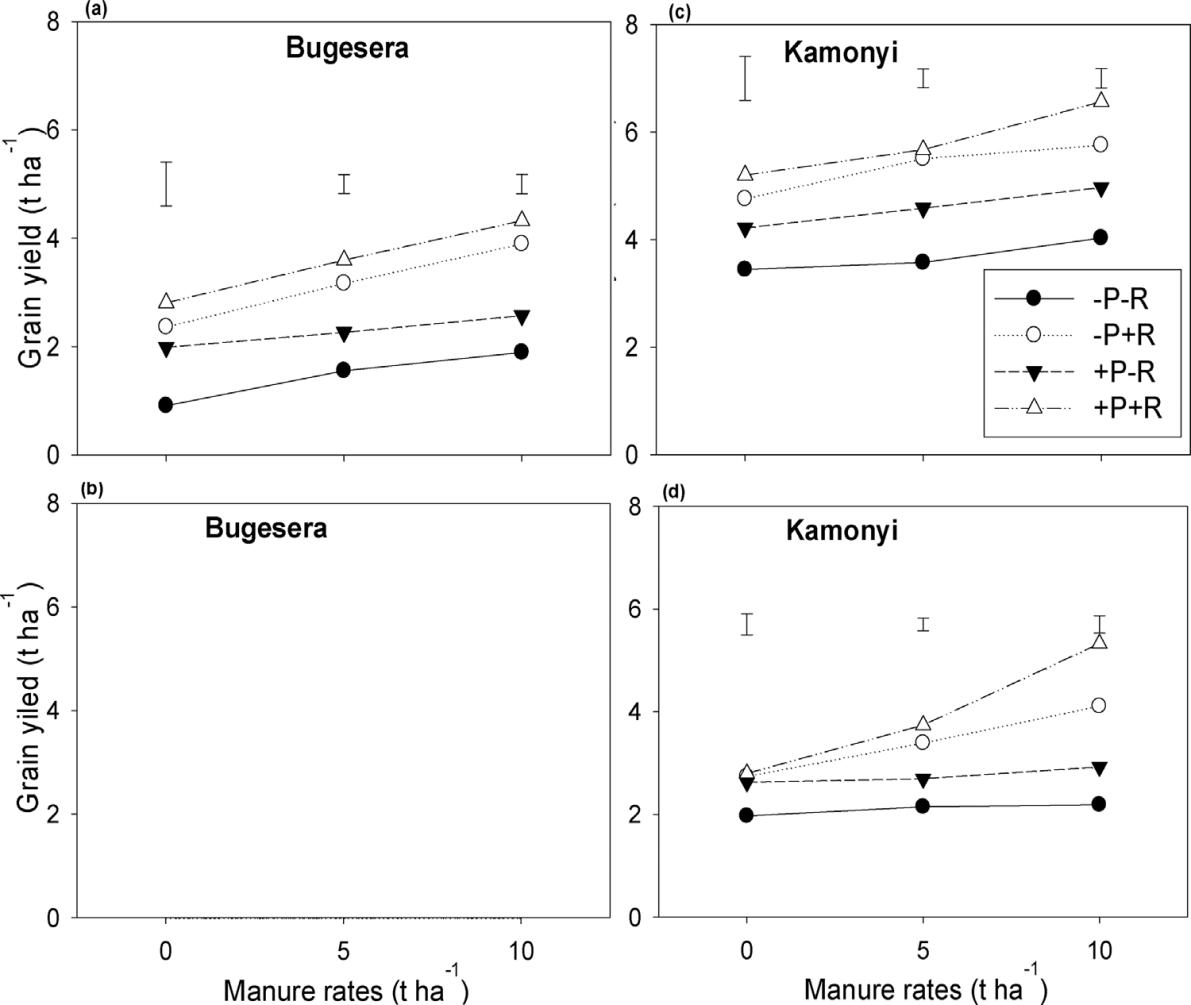
Maize grain yield after: (a, c) common bean and (b, d) soybean receiving different treatments at (a, b) Bugesera and (c, d) Kamonyi. Error bars represent the standard errors of difference between means; −/+ R: without or with rhizobia (R) inoculation. N.B. Maize failed to yield any grain after soybean at Bugesera.

Greater maize grain and stover yields after common bean were observed at Kamonyi (Fig. 5; Table 7). The late planting of maize due to the longer duration of the soybean crop at Bugesera, meant that the season ended before maize had yielded any grain. At Kamonyi, greater maize yield was achieved in treatments that previously received manure relative to unamended treatments or treatments with inoculation combined with P fertilizer. Whether maize was grown after common bean or soybean, synergistic effects of combined application of inputs applied to the two legumes were seen in increased yield of the maize. For instance, accumulated responses to the application of single inputs applied to the legume were 2.8 and 1.7 t ha^−1^ for maize grain yield grown after common bean and soybean respectively, whereas the combined effects of all inputs applied together were 3.0 and 3.4 t ha^−1^ for maize grain yield grown after common bean and soybean respectively. Similarly, when inoculation had been applied to the previous common bean, mean maize grain yield increased by 1.3 t ha^−1^ over maize yield after untreated common bean, and this response increased to 1.6 t ha^−1^ when manure and P fertilizer were added to inoculation. When manure had been applied to common bean, the maize grain yield increased by 0.7 t ha^−1^ over maize after unamended common bean, and the response increased by 1.3 t ha^−1^ when inoculation and 30 kg P ha^−1^ were added with manure in the legume phase. For maize grown after soybean, there was strong residual effect from inputs applied to soybean. For example in the case of maize grain yield grown after soybean, inoculation response to application of manure and P fertilizer increased from 0.8 to 2.4 t ha^−1^, and manure response to application of P fertilizer and inoculation increased from 0.2 to 2.5 t ha^−1^ (Fig. 5). Maize stover yield response to inputs applied to the preceding legumes followed similar pattern as grain yield (Table 7).

**Table 7:** Stover response of maize (t ha^−1^) grown after common bean and soybean with and without inoculation, P fertilizer and manure.

Treatments	Bugesera AEZ		Kamonyi AEZ	
	Common bean-Maize	Soybean-Maize	Common bean-Maize	Soybean-Maize
	Stover (t ha^−1^)	Stover (t ha^−1^)	Stover (t ha^−1^)	Stover (t ha^−1^)
0P-R + 0M	2.8	3.6	6.0	1.7
0P-R + 5M	3.5	2.9	6.6	1.8
0P-R + 10M	4.8	3.5	6.2	1.9
0P + R + 0M	6.0	4.2	6.4	2.3
0P + R + 5M	6.2	7.1	7.6	2.9
0P + R + 10M	5.8	7.3	7.3	3.5
30P-R + 0M	4.5	3.5	6.4	2.2
30P-R + 5M	5.1	4.1	7.0	2.3
30P-R + 10M	3.9	6.0	8.1	2.5
30P + R + 0M	3.7	5.9	5.8	2.4
30P + R + 5M	6.2	5.3	7.6	3.2
30P + R + 10M	7.7	8.0	7.6	4.6
Average	5.0	5.1	6.9	2.6
SED (Inoculum)	0.62	0.54		
SED (Manure)		0.59		0.50

## 4 Discussion

### 4.1 Growth and yields of common bean and soybean

The better and well distributed rainfall at Kamonyi (Fig. 1b) resulted in greater yields of stover and grain compared with Bugesera and Kayonza (Fig. 2, Fig. 3; Table 2). Despite the differences in overall productivity, the response of both legumes to the various treatments was remarkably consistent across the locations. Manure strongly increased the yield of both legumes. Inoculation and P fertilizer resulted in greater yield of both common bean and soybean in all three AEZs, and these treatment effects increased with increasing rate of manure applied. This suggests that manure contributed nutrients other than N and P or had other beneficial effects on growth of legumes (Zingore et al., 2008a, Zingore et al., 2008b). The individual effects from inoculation, P fertilizer or manure on increasing biomass at podding of common bean were clear, and effects were stronger when all inputs were combined suggesting synergy. Similar synergistic effects when different sources of nutrients are used together have been reported elsewhere (e.g. Mustonen et al., 2013). There was also evidence for synergistic effects of all inputs on soybean stover and grain yields but not for common bean where the effects of the inputs appeared to be additive.

### 4.2 Nodulation, N_2_-fixation, N and P uptake and net N input by the legumes in response to inputs

Greater nodulation was observed in soybean than common bean, and the largest nodule scores were seen in Kamonyi which received the most rainfall, well distributed through the season (Fig. 4). Inoculation significantly increased the nodule score of both legumes. Application of P fertilizer alone did not significantly enhance the nodule score of common bean but did for soybean. These differences in response to P fertilizer between the two legumes could be due to differences in P demand although the mechanisms behind are not well understood (Singh et al., 1997). The increase in nodulation observed with manure may be linked to its impacts on availability of other nutrients, on moisture supply or on better soil structure creating a more favourable environment for nodulation.

The better nodulation at Kamonyi was translated into a larger%Ndfa in both bean and soybean. There were no obvious or consistent effects of inoculation, P fertilizer or manure on%Ndfa. Similarly, Vandamme et al. (2014) observed that increasing P rates did not result in increased N_2_-fixation in different soybean genotypes in Kenya. The N available from manure is often depleted in ^15^N compared with the atmosphere (Inácio et al., 2015) which could result in overestimation of %Ndfa in the control treatments although we cannot assess how important this effect might have been. The smaller %Ndfa observed at Bugesera in both legumes could be due to water limitation resulting from dry spells and less total rainfall at this location.

The largest amounts of N_2_-fixed were achieved in both legumes when inoculation, P fertilizer and manure were applied together, reflecting the impacts on growth and yield at all three sites. The higher rainfall recorded at Kamonyi also resulted in a larger amount of N_2_-fixed than at Bugesera and Kayonza. Ojiem et al. (2007) also observed enhanced legume growth, biomass production and larger N_2_-fixation in agroecological zones with higher rainfall in Western Kenya.

The net N input from N_2_-fixation (calculated by subtracting the amount of N removed in grain from the amount of N_2_-fixed in each treatment) was negative in most cases for both common bean and soybean, with no consistent differences among treatments (Table 2, Table 3). Although treatments with the largest yields tended to have the most negative net inputs this was not always the case. The values were more negative where%Ndfa was smaller, especially at Bugesera and Kayonza the two sites which received least rainfall. Such results are not uncommon: there are many reports of negative net N inputs for grain legumes (e.g. Giller et al., 1994, Osunde et al., 2003). Net N inputs of grain legumes may be limited due to stresses such as drought that result in poor growth of the legume (Vanlauwe and Giller, 2006).

Greater shoot N and P uptake was observed at Kamonyi with higher rainfall and in treatments that received combined inoculation with manure and P fertilizer (Table 5). The greater N and P uptake in treatments that received manure relative to treatments with P fertilizer and inoculation, suggests that N and P supplied by manure also were readily available for plant uptake and positively contributed to plant growth.

### 4.3 Residual effect of legumes on maize

Maize grain and stover yields matched closely the pattern of biomass production and yield of the previous common bean and soybean treatment (Fig. 5; Table 7). The greatest residual effects of the legumes and largest maize yields were observed at Kamonyi with highest rainfall; matching observations of Ojiem et al. (2014) in western Kenya. Despite the fact that soybean fixed more N than common bean, its long duration led to the delay in sowing of the subsequent maize crop at Bugesera, resulting in complete failure to yield of the maize crop. These suggests the need for early maturing soybean varieties in Bugesera, or that farmers may be better to use common bean in rotation with maize. Inoculation along with manure and P fertilizer application to common bean and soybean significantly enhanced the yields of the subsequent maize crop. Application of inputs to the legumes also resulted in enhanced N and P uptake of the subsequent maize than that of maize after unamended legumes (Table 6). Whilst the residual benefits of P fertilizer and manure could be due to residual P availability (Wolf et al., 1987) and slow mineralization of the manure applied to the legumes, the increase in maize yield due to rhizobial inoculation can only be due to the improved growth and N_2_-fixation of the previous legume. Our results support the suggestions that inputs are best targeted to legumes in rotation with cereals to maximise legume production and N_2_-fixation as well as residual benefits to cereals (Giller, 2002).

We observed strong yield increases and N uptake of maize after common bean and soybean amended with inputs despite the negative net N input observed in some treatments. The increase of maize yield despite negative net N input also could be a result of another rotational effect rather than simply the carry-over of N from the soybean residue (Sanginga et al., 2001). Among these rotational effects include improvement of soil physical properties allowing better exploration of the soil by maize after the legumes (Sanginga et al., 2001), control of diseases and pests in cereals (Giller, 2001), high mycorrhizal infection and reduced incidence of nematode damage (Bagayoko et al., 2000). Nevertheless, the fact that legume inoculation also had strong residual benefits in production of the subsequent maize crop leads us to concur with Kermah et al. (2017) that the net N input from legumes is a poor indicator of effects of legumes on soil fertility.

### 4.4 Relevance of the study for rwandan smallholder farmers

Cultivation of legumes as sole crops is promoted through the government-led Crop Intensification Program (CIP) in Rwanda, with the recommendation that legumes are grown in rotation with cereals. Smallholder farmers largely grow legumes with little or no fertiliser. Most mineral or organic fertiliser is targeted to cash crops (e.g. tomato, vegetables, Irish potato) that have a ready market. Both common bean and soybean are important sources of income for farmers and our results show that if inputs are targeted to enhance legume production, greater yields are achieved both from the legume itself and the subsequent cereal crop. Although we compared manure applications of 5 or 10 t ha^−1^, the current recommendation in Rwanda is 10 t ha^−1^ for food crops (MINAGRI, 2010).

The question remains as to whether these are feasible rates of manure for smallholders? To answer this question, we need to consider livestock numbers, livestock ownership and farmers’ resources. In recent years, the government of Rwanda has promoted ownership of improved dairy cows and zero grazing through the “One Cow per Poor Family” programme. This programme targets poor and vulnerable farmers with no cattle and less than 0.75 ha of land, on condition that the first offspring is passed on to another farmer (MINAGRI, 2009). This programme is expected to improve livelihoods through consumption and sales of milk and increased crop productivity through use of manure. A remarkable increase in the size of the national herd has been observed rising to 1.33 million cattle in 2013 from 755,000 in 2000, with the projects of 1.67 million in 2017 and 1.92 million by 2020 (MINAGRI, 2013).

There are 2.41 million households in rural Rwanda of which 32% own cattle (NISR, 2014); so 771,200 households own 1,33 million cattle, giving an average 1.7 cattle hh^−1^. It has been shown that a local Ankole weighing 300 kg, reared in a zero-grazing system can produce 6 t of manure per year (MINAGRI, 1990). Assuming each head cattle produces 6 t of manure annually each household could produce on average 10.2 t of manure per year. Landholdings in Rwanda are generally very small but vary from one region to another. At national level more than 60% of households have less than 0.7 ha. Some districts are densely-populated such as Kamonyi which has a population density of 519 inhabitants/km^2^. By contrast, Bugesera district has 280 inhabitants/km^2^ and Kayonza is the least populated with 178 inhabitants/km^2^ (NISR, 2014). In all three districts average farm size is reported to be 0.5–1 ha per household. The above calculations suggest it is feasible for farmers who own at least one head of cattle to apply manure at rates of 5 to 10 t ha^−1^ per year.

Bucagu et al. (2014) working in Isimbi (Southern province) and Kageyo (Northern province) of Rwanda showed that farmers with 0.5 to 2 ha of lands and one or two head of cattle were able to invest organic manure in food crop production. In northern Rwanda, Franke et al. (2016) found that poor farmers were cultivating on poorly fertile soils and achieved poor yields of climbing beans, while wealthier farmers could invest in organic and inorganic fertiliser and achieve greater yields. Analysis in southwest Rwanda suggests that limited fodder availability at village scale may limit the expansion of the One Cow per Poor Family programme to all households (Klapwijk et al., 2014). Although some farmers bought manure for growing tomatoes and vegetables in our study sites, there was no fixed price. In Kamonyi, a pit of approximately 5 t manure was sold for 20,000 Rwandan Francs (€24), whereas in Bugesera it was only 10,000 Rwandan Francs (€12) and Kayonza between 15,000–20,000 Rwandan Francs. It is unlikely that the poorer farmers would be able to invest in purchasing manure for crop production.

## 5 Conclusion

The use of inoculum combined with manure and P fertilizer on common bean and soybean showed great potential to enhance not only the yields of the legume but also production of the subsequent maize crop. In the drier agroecology of Bugesera, maize failed to yield any grain when grown after soybean due to the delayed planting due to the long duration of soybean compared with common bean. Early maturing soybean varieties are required in such regions of low and erratic rainfall in case of soybean-maize rotations. Our results also show strong influence of the agroecological environment and call for careful strategies when targeting technologies and crops for sustainable intensification of crop production.
